# Effects of the mode of re-socialization after juvenile social isolation on medial prefrontal cortex myelination and function

**DOI:** 10.1038/s41598-017-05632-2

**Published:** 2017-07-14

**Authors:** Manabu Makinodan, Daisuke Ikawa, Kazuhiko Yamamuro, Yasunori Yamashita, Michihiro Toritsuka, Sohei Kimoto, Takahira Yamauchi, Kazuki Okumura, Takashi Komori, Shin-ichi Fukami, Hiroki Yoshino, Shigenobu Kanba, Akio Wanaka, Toshifumi Kishimoto

**Affiliations:** 1 Department of Psychiatry, Nara Medical University School of Medicine, Nara, Japan; 2 Department of Anatomy and Neuroscience, Nara Medical University School of Medicine, Nara, Japan; 3 Department of Neuropsychiatry, Graduate School of Medical Science, Kyusyu University, Fukuoka, Japan

## Abstract

Social isolation is an important factor in the development of psychiatric disorders. It is necessary to develop an effective psychological treatment, such as cognitive rehabilitation, for children who have already suffered from social isolation, such as neglect and social rejection. We used socially isolated mice to validate whether elaborate re-socialization after juvenile social isolation can restore hypomyelination in the medial prefrontal cortex (mPFC) and the attendant functions manifested in socially isolated mice. While mice who underwent re-socialization with socially isolated mice after juvenile social isolation (Re-IS mice) demonstrated less mPFC activity during exposure to a strange mouse, as well as thinner myelin in the mPFC than controls, mice who underwent re-socialization with socially housed mice after juvenile social isolation (Re-SH mice) caught up with the controls in terms of most mPFC functions, as well as myelination. Moreover, social interaction of Re-IS mice was reduced as compared to controls, but Re-SH mice showed an amount of social interaction comparable to that of controls. These results suggest that the mode of re-socialization after juvenile social isolation has significant effects on myelination in the mPFC and the attendant functions in mice, indicating the importance of appropriate psychosocial intervention after social isolation.

## Introduction

Multiple lines of evidence have shown that social experience during the juvenile period substantially affects brain function and behavior in rodents, dogs, and humans^1–4^. Interestingly, juvenile social experience changes not only brain function, but also brain structure, by affecting myelination^5–7^. A number of studies have indicated that aberrant juvenile social experiences have long-lasting effects on brain function and structure, extending into adulthood in rodents^6, 8, 9^ and humans^5, 10^. In addition, other studies have suggested that interventions, such as re-socialization after social isolation, and an enriched environment after adverse juvenile experience can recover these brain abnormalities in rodents^7, 11^ and humans^12^.

Social isolation, such as neglect and social rejection, which is a form of social experience shutdown in terms of experiencing being disliked or not disliked^13^, is a key factor in the development or exacerbation of psychiatric disorders, such as mood disorders, anxiety disorders, personality disorders, attention-deficit hyperactivity disorder, and autism spectrum disorder (ASD)^14–16^. While the most important intervention is to prevent neglect^17^, it is also necessary to develop an effective treatment for children who have already suffered from social isolation. Recently, psychosocial interventions, such as cognitive behavioral therapy, have attracted increasing attention in clinical psychiatry^18, 19^, even for patients with conditions that are regarded as refractory or involving permanent disability, e.g., schizophrenia and ASD, as well as for abused children^20^. Whereas the main purpose of psychosocial interventions is to improve brain function, recent studies have suggested that such interventions change both brain structure and function^21, 22^.

**Figure 1:**
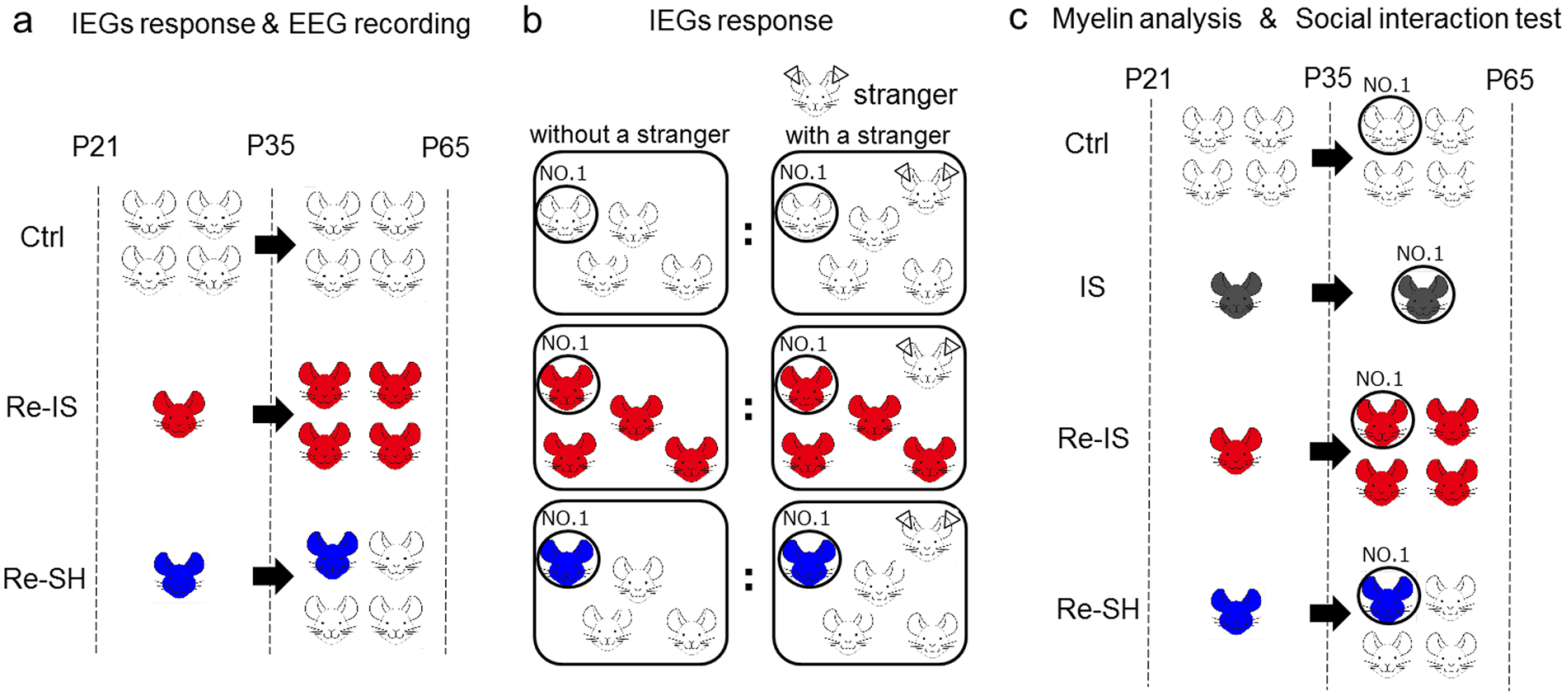
Experimental designs.

Recent studies have shown that myelination in the central nervous system is essential for acquiring motor skills and that myelination in the medial prefrontal cortex (mPFC) is necessary for sociability in mice^23, 24^. These findings indicate that myelination in the mPFC can be one of the critical elements in mPFC functions. A previous study has shown that re-socialization with socially isolated mice does not alter the hypomyelination in the mPFC caused by juvenile social isolation^6^, which indicates that social interaction between socially isolated mice is not sufficient to restore hypomyelination. In this study, we employed socially isolated mice as a model of neglect and social rejection and sought to investigate whether re-socialization with socially housed mice, rather than with socially isolated mice, after juvenile social isolation (Fig. 1), can restore hypomyelination in the mPFC and the attendant brain dysfunction, even after myelin impairment is manifest in mice due to juvenile social isolation.

## Materials and Methods

### Mice and housing conditions

Male C57BL/6J mice were used for each experiment and there was no duplication of mice between experiments. Mice were maintained in a fixed 12-h light-dark cycle (light period, 8:00−20:00; dark period, 20:00−8:00). After weaning at postnatal day 21 (P21), four littermates were reared together in a cage until P65 (control, Ctrl mice) or a mouse was housed in a cage until P35 or P65 (isolation, IS mice). Then, each isolated mouse for 2 weeks after weaning was housed together with three other male isolated mice from the same litter (re-socialization) in a cage, from P35 until P65 (re-socialization with isolated mice; Re-IS mice). On the other hand, from P21 until P35, one mouse was housed individually and three other mice from the same litter were socially housed, and these four mice were then housed together in a cage from P35 until P65 (re-socialization with socially housed mice; Re-SH mice) (Fig. 1a,c). Experimental protocols were approved by the Animal Care Committee of Nara Medical University and all methods were carried out in accordance with the policies of the National Institutes of Health Guide for the Care and Use of Laboratory Animals.

### Response of IEGs in the mPFC to social exposure

Experimental pairs of mice (P65) were set up for monitoring IEG mRNA expression in experimental mice in the presence and absence of a strange mouse (that was socially housed) in order to measure neuronal activity in the mPFC during social interaction. A male age-matched mouse was placed into a cage in which four male age-matched experimental littermates (Ctrl, Re-IS, or Re-SH) were housed, and 30 min later, each randomly numbered experimental mouse (No. 1–4) was decapitated to measure IEG mRNA expression in the mPFC (“IEG mRNA expression with exposure”). Alternatively, four co-housed Ctrl, Re-IS, or Re-SH mice (No. 1–4) were decapitated to measure IEG mRNA expression in the mPFC (“IEG mRNA expression without exposure”). The ratio of mPFC neuronal activity (IEG mRNA expression with exposure/IEG mRNA expression without exposure) of randomly paired mice (No. 1 from mice with exposure and No. 1 from mice without exposure) was measured. This experiment was performed in dim light during the dark period.

### Quantitative reverse transcription polymerase chain reaction (qRT-PCR)

mRNA levels of *cFos*, *Arc*, and *Npas4* were measured in layers V and VI of the mouse mPFC at P65 by means of qRT-PCR. The details have previously been described^6^. Coronal sections (1-mm-thick) of layer V and VI in the prelimbic and infralimbic areas of the mPFC were dissected around Bregma 1.70 mm^25^. RNA was isolated using RNeasy Mini Kit (Qiagen; Valencia, CA, USA) following the manufacturer’s protocol, and any genomic DNA contamination was eliminated by DNase treatment. RNA quantity was determined by absorbance at 260 nm. cDNA was then synthesized from 500 ng of RNA using an iScript kit (Bio-Rad Laboratories; Hercules, CA, USA) as recommended by the supplier. qRT-PCR was performed using SYBR *Premix Ex Taq* ^TM^ II (Takara Bio Inc., Shiga, Japan) according to the manufacturer’s recommendations. The 18S ribosomal RNA was chosen as an internal control for the delta-delta C_T_ method. Primer sequences are available upon request.

### *In vivo* electroencephalogram (EEG)

In order to measure δ- (0.5–4 Hz), θ- (4–8 Hz), α- (8–12 Hz), σ- (12–16 Hz), β- (16–24 Hz), and γ- (25–55 Hz) oscillations in the mPFC, we used a telemetry implant (PhysioTel^®^ transmitter TA10ETA-F20; DSI, Lexington, KY, USA) and a receiver (RPC-1, DSI). Mice were anesthetized with chloral hydrate (400 mg/kg) (Nacalai Tesque Inc., Kyoto, Japan). An electric probe was stereotaxically inserted into layer V within the prelimbic and infralimbic areas of mPFC (Bregma: 1.9 mm anterior, 0.5 mm lateral [right], 3.5 mm ventral) and the other electrode was placed in the longitudinal cerebral fissure (Bregma: 6 mm posterior, 0-mm lateral). Dataquest^TM^ A.R.T.^TM^ (DSI) software was used to acquire and analyze EEG data. EEGs were recorded during the habituation period, in which mice were placed in a novel cage for 30 min, and during subsequent social interaction, in which an age-matched male mouse was inserted into the cage for 5 min. The ratio of oscillation power during social interaction (per 5 min) to the average of oscillation power during habituation (per 5 min) was measured to evaluate mPFC function in response to social interaction. The randomization of mice for examination was performed as in the IEG response experiments (Fig. 1b). This experiment was performed in darkness during the dark period.

### Electron microscopy

Four mice per condition (Ctrl, IS, Re-IS, and Re-SH), at P65, were perfused intracardially with fixative (2% paraformaldehyde, 2.5% glutaraldehyde, and 0.1% picric acid in 100 mM cacodylate buffer); the brains were removed from the skulls and fixed in the same solution overnight. Coronal sections (500-µm-thick) were then obtained to isolate the mPFC (between Bregma 1.70 mm and 1.98 mm), embedded in epoxy resin, and examined by transmission electron microscope in 50-nm sections. Using this approach, multiple, non-overlapping regions of the brain from each mouse were imaged. Three mice were excluded from the study due to insufficient myelin fixation. A g-ratio was calculated as the axon perimeter/(axon + myelin sheath) perimeter, and axons with circularity (4 × π × area/perimeter^2^) under 6.0 were excluded from analysis, because the perimeter does not precisely reflect myelin thickness in cases where the axons were cut at a slant. The average value of the g-ratio from each mouse was used for statistical analysis.

### Social interaction test

Two clear perforated boxes, one of which contained a male mouse at about P120, and which had not been housed with experimental mice, were placed in an open field (40 cm × 40 cm). The Ctrl, IS, Re-IS, and Re-SH mice were placed in the open field and was allowed to explore the apparatus freely for 5 min. A video tracking system traced the nose point and center point of their bodies to measure social interaction and locomotion, respectively, and then the time spent within 2.5 cm of each box was recorded (TopScan Suite, Clever Sys Inc., Reston, VA, USA). Social interaction was analyzed as the ratio of time spent exploring the box containing the mouse to the total time approaching both boxes. This experiment was performed in dim light during the dark period.

### Statistical analyses

Analysis of variance (ANOVA) followed by the Newman-Keuls multiple comparison test was used for statistical analyses with GraphPad Prism. Data are presented as mean ± standard error of mean. P values less than 0.05 were considered statistically significant.

## Results

### The mode of re-socialization after juvenile social isolation affects mPFC activity

**Figure 2:**
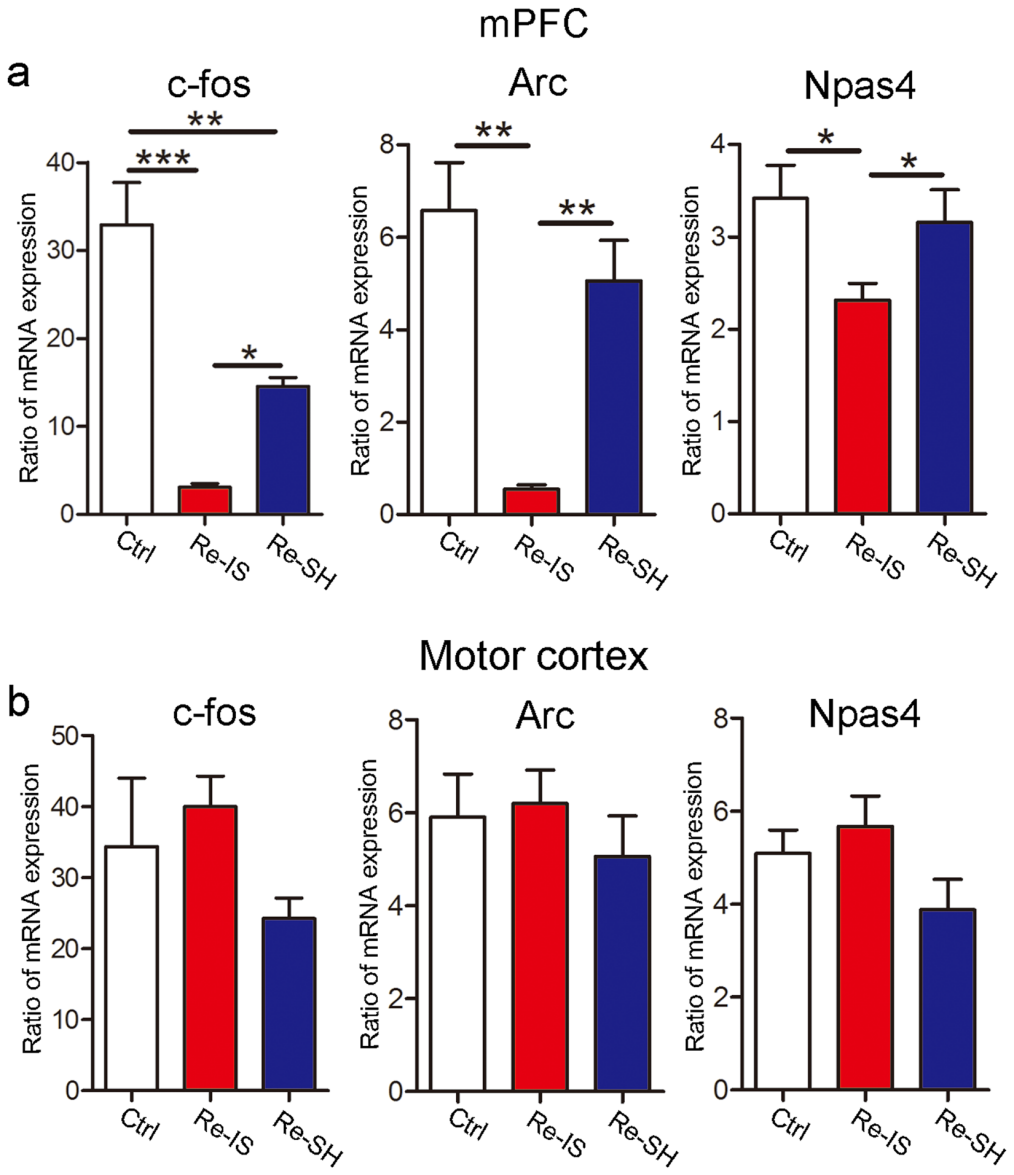
Immediate early gene responses in the medial prefrontal cortex (mPFC) depend on the mode of re-socialization. (**a**,**b**) Ratio of immediate early gene (IEG) expression 30 min after exposure to a strange mouse (IEG mRNA expression with exposure/IEG mRNA expression without exposure) in the mPFC and motor cortex. (**a**) While an increase in *cFos* expression caused by exposure to a strange mouse was significantly decreased in both Re-IS and Re-SH mice, as compared to Ctrl mice, Re-SH mice showed a higher *cFos* response than Re-IS mice. The response of *Arc* and *Npas4* in Re-IS mice was lower than that of Ctrl and Re-SH mice, and Ctrl and Re-SH mice showed a comparable response. (**b**) In the motor cortex, there were no differences in terms of changes in IEG expression after exposure to a strange mouse. n = 4/group, *p < 0.05, **p < 0.01, ***p < 0.001. Error bars = SEM.

Since hypoactive mPFC function after child maltreatment has been reported^26^, we hypothesized that mPFC function would be affected by the mode of re-socialization. Immediate early gene (IEG) responses in the mPFC during exposure to a strange mouse were measured as an index of mPFC function (IEG mRNA expression with exposure/IEG mRNA expression without exposure). In this study, we employed *cFos* as a general marker of neuronal activity^27^, *Arc* as a marker of excitatory synapse^28^, and *Npas4* as a marker of inhibitory synapse^29^. As expected, changes in *cFos*, *Arc*, and *Npas4* expression were much less marked in Re-IS mice than in Ctrl mice, but were not significantly different between Ctrl mice and Re-SH mice, except for that of *cFos*, while Re-SH mice showed higher expression levels of these genes than did Re-IS mice (*cFos*, n = 4/group, F(2, 9) = 28.34; *Arc*, n = 4/group, F(2, 9) = 16.01; *Npas4*, n = 4/group, F(2, 9) = 4.878) (Fig. 2a). On the other hand, there were no differences in terms of changes in IEG expression after exposure to a strange mouse in the motor cortex (*cFos*, n = 4/group, F(2, 9) = 1.650; *Arc*, n = 4/group, F(2, 9) = 0.4589; *Npas4*, n = 4/group, F(2, 9) = 2.344) (Fig. 2b). These results suggest that the mode of re-socialization after juvenile social isolation affects mPFC activity during the exposure to a strange mouse.

### The mode of re-socialization after juvenile social isolation affects the power of γ-oscillation and β-oscillation

**Figure 3:**
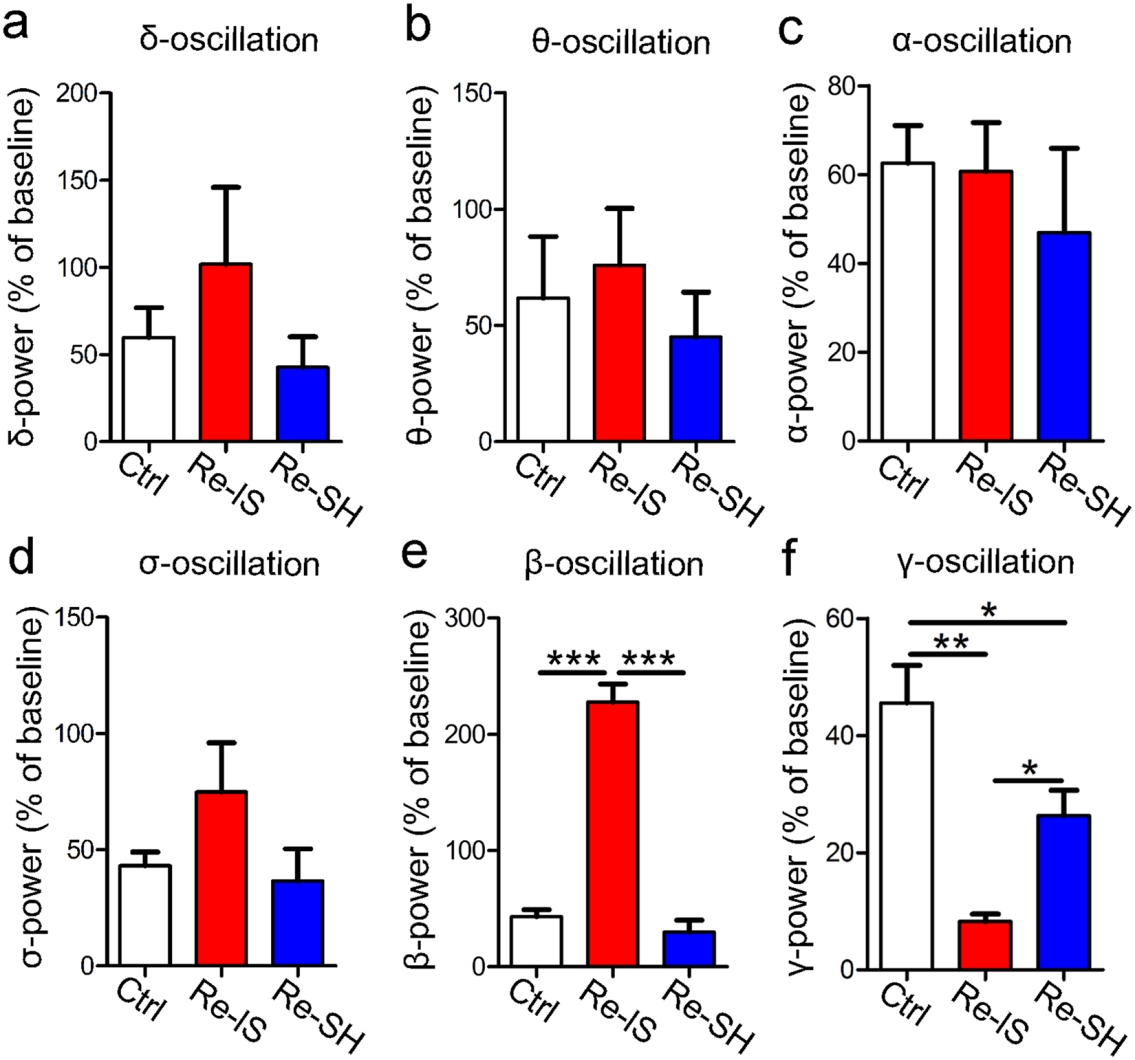
The powers of β- and γ-oscillation in the medial prefrontal cortex (mPFC) are dependent on the mode of re-socialization. The powers of δ-, θ-, α-, σ-, β-, and γ-oscillation in the m PFC during exposure to a strange mouse for 5 min (relative to baseline) were measured. (**a**–**d**) The power of either δ-, θ-, α-, or σ-oscillation was not different between Ctrl, Re-IS, and Re-SH mice. (**e**) The power of β-oscillation was markedly increased in Re-IS mice as compared to Ctrl and Re-SH mice. (**f**) The increase in γ-oscillation power in Re-IS mice was significantly less than that in Ctrl and Re-SH mice, while the amount of change in Re-SH mice was less than that in Ctrl mice. n = 3/group, *p < 0.05, **p < 0.01, ***p < 0.01. Error bars = SEM.

Based on these findings, we further aimed to investigate mPFC function by evaluating changes in the power of electric oscillations (δ, θ, α, σ, β, γ) in the mPFC in response to a strange mouse, which could be an electrophysiological measure for social interaction^30^. Interestingly, the responses in γ-oscillation paralleled expression of one of the IEGs (*cFos*). Re-IS mice had lower responses in γ-oscillation than did Ctrl mice and Re-SH mice, while Re-SH mice had lower responses than Ctrl mice (n = 3/group, F(2, 6) = 17.21) (Fig. 3f). In contrast, the power of β-oscillation was markedly higher in Re-IS mice than in Ctrl and Re-SH mice (n = 3/group, F(2, 6) = 96.68) (Fig. 3e). The powers of δ-, θ-, α-, σ-oscillations were not different between the three groups (n = 3/group, F(2, 6) = 1.100; n = 3/group, F(2, 6) = 0.4345; n = 3/group, F(2, 6) = 0.3958; n = 3/group, F(2, 6) = 1.890), respectively) (Fig. 3a–d).

### The mode of re-socialization after juvenile social isolation affects myelination in the mPFC

**Figure 4:**
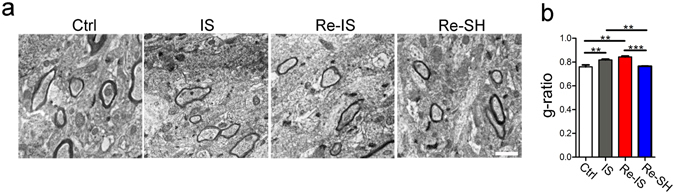
Myelin thickness in the medial prefrontal cortex (mPFC) depends on the mode of re-socialization. (**a**) Images of myelinated axons in layer V of the mPFC. (**b**) The average g-ratio of axons from each mouse (n = 3) was used as a statistical value. The statistical comparison of Ctrl, IS, Re-IS, and Re-SH mice showed that the myelin of IS and Re-IS mice was thinner than that of Ctrl and Re-SH mice, while Ctrl and Re-SH mice showed comparable myelin thickness (more than 50 axons/each mouse, n = 3/group), **p < 0.01, ***p < 0.001, scale bar = 0.5 μm. Error bars = SEM.

In order to evaluate whether the mode of re-socialization after juvenile social isolation affected myelination in the mPFC, an index of myelin thickness, the g-ratio, in layer V of the mPFC, was compared. We statistically compared the average g-ratio of more than 50 axons from each mouse (n = 3/group). While IS and Re-IS mice had thinner myelin in the mPFC than did Ctrl mice, Re-SH mice had a myelin thickness comparable to that of Ctrl mice (F(3, 8) = 19.29) (Fig. 4a,b), indicating that myelination in the mPFC is sensitive to the mode of re-socialization after juvenile social isolation.

### The mode of re-socialization after juvenile social isolation affects social interaction

**Figure 5:**
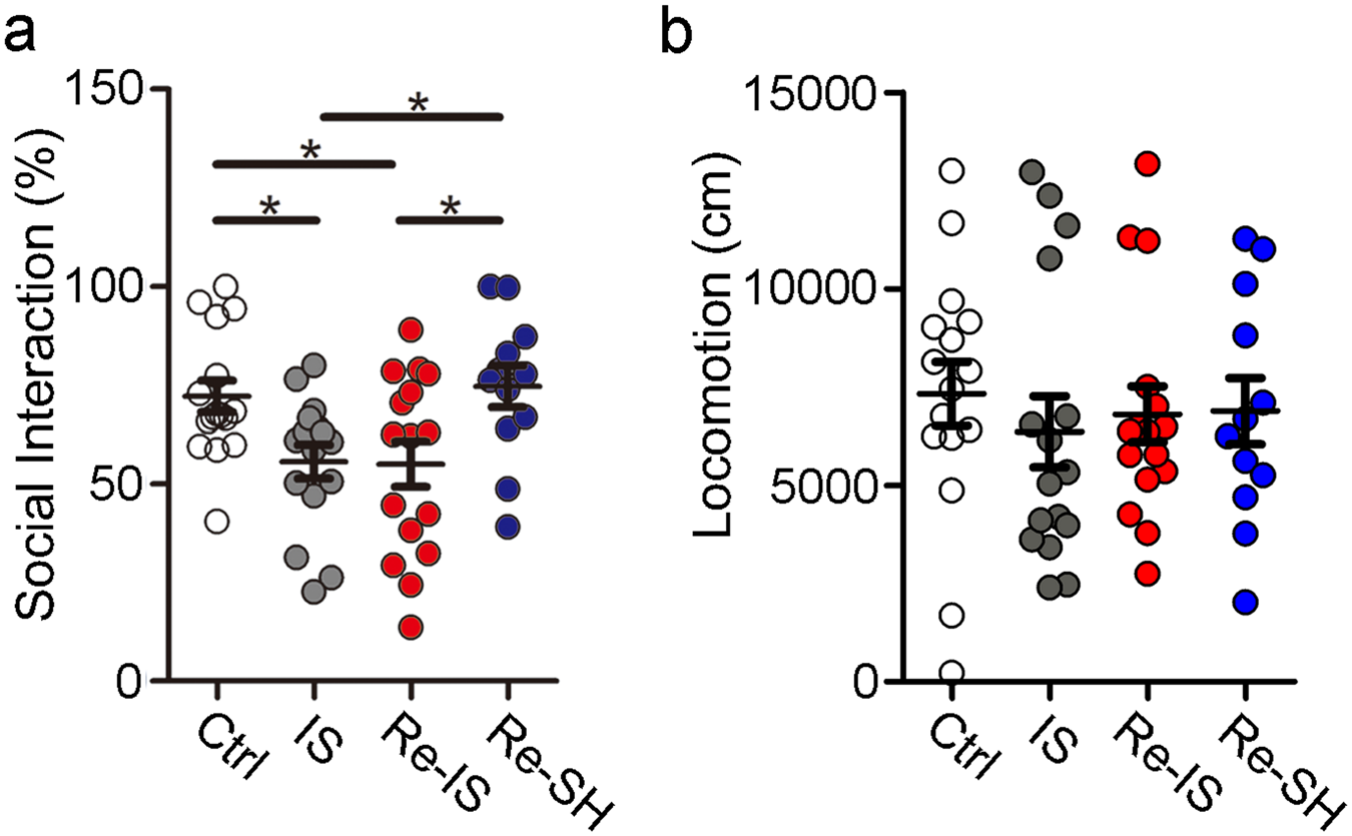
Social interaction depends on the mode of re-socialization. The ratio of time taken to approach a box with a mouse to that taken to approach a box without a mouse was measured. (**a**) Social interaction of IS and Re-IS mice was reduced compared to that of Ctrl, while Ctrl and Re-SH mice showed a comparable score for social interaction. Re-SH mice showed higher social interaction than IS and Re-IS mice. (**b**) There were no differences in the distance of locomotion during social interaction test. Ctrl; n = 16, IS; n = 16, Re-IS; n = 16, Re-SH; n = 12, *p < 0.05. Error bars = SEM.

A recent study has shown that restoration of myelination in the mPFC can improve impaired social interaction of mice exposed to protracted social isolation^24^, which suggests that myelin can control social interaction. In the current study, IS and Re-IS mice with hypomyelination in the mPFC showed poor social interaction as compared to Ctrl mice with intact myelination, while Re-SH mice with intact myelination demonstrated social interaction comparable to that of Ctrl mice. Re-SH mice showed higher social interaction than IS and Re-IS mice (Ctrl, n = 16; IS, n = 16; Re-IS, n = 16; Re-SH, n = 12, F(3, 56) = 4.558) (Fig. 5a). There were no differences in the locomotive distance of mice during the social interaction test (Ctrl, n = 16; IS, n = 16; Re-IS, n = 16; Re-SH, n = 12, F(3, 56) = 0.2461) (Fig. 5b). These results indicated a possibility that appropriate re-socialization among others, after juvenile social isolation, can improve social interaction by restoring myelination.

## Discussion

Neglect affects children devastatingly and deeply, and possibly influences their mental state for the rest of their life. Children suffering neglect often present with language deficits, which may be due to insufficient language exposure to others. The language problem may exaggerate the extent of their social isolation. Those children are also likely to have mental problems, such as anxiety, depression, somatization, paranoia, and hostility^31^. Based on these clinical studies, we investigated whether psychosocial interventions after juvenile social isolation, leading to hypomyelination, could affect myelination in the mPFC and the attendant functions, whereas it remains unclear whether social isolation for 14 days can mimic neglect or social rejection of human beings. In our study, a mouse was socially isolated during the juvenile period and then re-socialized with its three littermates that had also been socially isolated. Considering that the behaviors of socially isolated mice appear abnormal, we considered that re-socialization with other socially isolated mice would be inappropriate as a sole method for validating the effects of re-socialization. Therefore, in the current study, we also isolated a mouse during the juvenile period and then placed it back with three littermates that had been socially housed. We then investigated the phenotypes created by these seemingly subtle differences in social interaction.

While social interaction between socially isolated mice did not change myelination, re-socializing these mice with socially housed mice resulted in restoration of hypomyelination in the mPFC, as well as recovery of mPFC dysfunctions caused by juvenile social isolation from P21 to P35. In contrast to the mPFC, social experience had no effects on locomotor function (Fig. 5b) or myelination in the motor cortex^6^, indicative of a lack of a motor function response as well as myelination in the motor cortex to social experience. These findings may indicate that appropriate interventions can be beneficial, even after brain structure has been damaged by juvenile social isolation, implying that psychosocial approaches can be effective for the treatment of abused children, and can even affect aspects of brain structure, such as myelination, in the mPFC.

Myelin speeds up the axonal conduction velocity by inducing saltatory conduction^32^. If myelination is impaired, and axonal conduction velocity consequently altered, the brain function in the affected region is likely to be disordered due to loss of optimal synchrony of the neuronal networks^33^. In this study, we focused on myelination in layer V of the mPFC, because myelination is much more substantial in layers V and VI than in layers II, III, and IV^34^. The differences in myelination may explain the variations in the IEG responses and in γ-oscillation in layers V and VI of the mPFC, between Re-IS mice and Re-SH mice. Several reports have provided evidence that myelin impairment may be a primary cause of psychiatric symptoms. Transgenic mice with a primary oligodendrocyte deficit show schizophrenia-like symptoms^35^, while the restoration of social isolation-induced hypomyelination improves social interaction ability^24^. Taken together, our findings indicate that the reduced sociability of Re-IS mice may be caused by hypomyelination in the mPFC.

On the other hand, it has also been shown that neuronal activity changes myelination^36^. Oligodendrocytes express membrane receptors for various growth factors and, interestingly, also express ion channels and neurotransmitter receptors. In addition to growth factors, adenosine tyrosine kinase, adenosine, gamma-aminobutyric acid, and glutamate have been shown to address oligodendrocytes and affect myelination^33, 36, 37^. Neurotransmitters, such as glutamate, have traditionally been regarded as molecules that are released at synapses. However, recent studies have shown that neurotransmitters can also be released from axons through membrane channels^37^. Therefore, oligodendrocytes can directly receive such molecules from axons, forming activity-dependent myelination along the axons. Based on these findings, the restoration of mPFC myelination in Re-SH mice can possibly be attributed to high neuronal activities in the mPFC as compared to Re-IS mice, as shown in this study. Furthermore, a recent article has also indicated that the extent of γ-oscillation might be markedly subject to activity-dependent myelination^38^, suggesting that myelination may shape neural networks.

In the current study, two types of measurements were employed to investigate mPFC function, viz., IEG responses and γ-oscillations. We chose to investigate *cFos*, *Arc*, and *Npas4* among the IEGs. *cFos* mRNA expression acts as a general marker of recent neuronal activity, as *cFos* expression is elevated immediately after neuronal activation^39^. The levels of *Arc* and *Npas4* mRNA are markers of excitatory and inhibitory transmission in neurons, respectively^40, 41^, and they are also critical molecules for the formation of excitatory and inhibitory synapses, respectively. Since the expression of all these three IEGs in Ctrl and RE-SH mice was higher than that in Re-IS mice, both excitatory and inhibitory transmission in neurons were apparently increased by re-socialization with socially housed mice as compared to social isolated mice. On the other hand, γ-oscillation is a rhythmic γ-band neural activity, and in the mPFC, γ-oscillation has been shown to play a critical role in maintaining the excitatory-inhibitory balance (E-I balance) that is related to emotion, cognition, and social interaction^30, 42, 43^. γ-Oscillation is controlled by parvalbumin-positive cells, which are inhibitory neurons^42^. Our current results suggest that re-socialization with socially housed mice may increase the function of the parvalbumin-positive cells in the mPFC, which may alter the E-I balance and thereby social interaction. The elevated function of the parvalbumin-positive cells may accelerate axonal activity-dependent myelination on the basis of recent finding that axons of inhibitory neurons are substantially myelinated^34^. Further electrophysiological and histological experiments are needed to dissect the E-I balance and myelination in the mPFC after re-socialization.

Both β- and γ-oscillation in the mPFC are related to attention; top-down attention tasks are mediated by β-oscillation and the power of γ-oscillation increases during bottom-up attention tasks^44, 45^. Based on these findings, Re-IS mice and Re-SH mice might have different modalities of attention due to altered frequencies of oscillations; this may underlie our social interaction test findings. Whereas the mechanism underlying the switches between β- and γ-oscillation in the mPFC remains unknown, recent studies have shown that the competition between these oscillations depends on sensory inputs and centrifugal inputs in the olfactory bulb. This indicates that different natural conditions, such as odor features and behaviors, affect the occurrence of both oscillations^46^. Based on these findings, we speculate that different inputs into the mPFC between Re-IS and Re-SH mice might control β- and γ-oscillation in the brain region. Although it is interesting to investigate oscillations of IS mice as a reference to Re-IS and Re-SH mice, it is not possible to examine mPFC responses to social exposure of IS mice after the current experimental protocol, in which four littermates in a home cage were exposed to a strange mouse; this protocol is not applicable to IS mice individually housed in a cage.

Altogether, we found that the two types of re-socialization led to different neuronal activities and myelination in the mPFC; nevertheless, further studies are needed to observe the exact differences in social interaction during re-socialization. We speculated that the amount of social interaction between isolated mice (Re-IS) was less than that between the isolated mice and socially housed mice (Re-SH). Based on the current findings, it is reasonable to consider that the differences in myelin formation are dependent on neuronal activities in this brain region, although it is not possible to deduce whether myelination or neuronal activity occurred first. As exercise accelerates myelination and new myelination is necessary to acquire exercise skill^23^, we hypothesize that, analogously, more “intense” re-socialization after juvenile social isolation may accelerate myelination in the mPFC, and that this novel myelination is necessary for acquiring social skills. These findings strongly support the possibility that an appropriate psychosocial approach can be used as intervention for abused children, even when they have already developed psychiatric symptoms, as it alters myelination and the attendant functions in the mPFC. This study warrants further investigations to assess whether changes in myelination or neuronal function is the primary cause of the impaired social interaction that is caused by social isolation of juveniles.
